# Structural and Release Properties of Combined Curcumin Controlled-Release Tablets Formulated with Chitosan/Sodium Alginate/HPMC

**DOI:** 10.3390/foods13132022

**Published:** 2024-06-26

**Authors:** Jing-Ting Lin, Yi-Chan Chiang, Po-Hsien Li, Po-Yuan Chiang

**Affiliations:** 1 Department of Food Science and Biotechnology, National Chung Hsing University, Taichung 40227, Taiwan; 2 Department of Food and Nutrition, Providence University, Taichung 43301, Taiwan

**Keywords:** curcumin, combined controlled-release tablet, simulated gastrointestinal tract

## Abstract

Controlled-release tablets offer several benefits, such as controlled release, odor masking, ease of use, stability, extended shelf life, and reduced production costs. This study developed combined curcumin controlled-release tablets (CCCTs) to increase the bioavailability of curcumin with hydroxypropyl methylcellulose (HPMC), chitosan, and sodium alginate. The hardness of the CCCTs was 5.63–1.98 kgf, friability was 0.00–1.22%, and disintegration time was 0.00–401.25 min. Differential scanning calorimetry and Fourier-transform infrared spectroscopy indicated a high compatibility between the excipients and curcumin. CCCTs with chitosan formed a gel structure, impeded disintegration, and reduced the release rate to 72.5% in simulated gastric fluid. In simulated intestinal fluid, CCCT with the HPMC–sodium alginate group formed a polyelectrolyte membrane hydrogel to prolong release from 6 to 12 h. This study developed various CCCT formulations that can be delivered through the gastric or intestinal tracts, using chitosan and HPMC–sodium alginate as excipients, respectively. CCCT can be used as a reference strategy for controlled-release curcumin delivery in the functional and healthcare supplement development.

## 1. Introduction

In recent years, the health supplement market has experienced significant growth, from 320 billion dollars in 2020 to 352.92 billion dollars in 2021 [1]. This growth has been attributed to an increasing emphasis on dietary interventions to prevent chronic diseases and promote overall health [2,3]. Various healthcare supplements, such as tablets, capsules, and powders, were applied for active pharmaceutical ingredient (API) delivery [1,4]. Tablets are often chosen as carriers because of their advantages of efficient mass production, convenient consumption, and stability during storage and transportation. However, these systems have some limitations, such as the need for frequent and multiple administrations and the rapid release of API, resulting in an abrupt surge in their concentration within the bloodstream and inducing physiological discomfort. Controlled-release systems are commonly employed in the formulation of health supplements, including tablets and capsules. In our recent study, the anthocyanin from purple sweet potato extracts was developed as gastric floating tablets with controlled-release gastric fluid efficiency [1]. To improve bioavailability, the adjusting of tablet formulation can be flexible for controlled release in gastric or intestinal fluids. However, the development is restricted by the overall flow characteristics and uniformity. For this, excipients play an important role and are classified based on their properties, such as diluents, binders, disintegrants, and lubricants [5,6].

Hydrogels are utilized in these systems as excipients to control the release of API, such as hydroxypropyl methylcellulose (HPMC), chitosan, and sodium alginate, while starch is often used as a disintegrant. These excipients were known for their ability to control the release of API in tablet formulations. HPMC, a neutral polymer derived from cellulose, exhibited remarkable stability over a pH range of 3 to 11 and formed a cross-linked gel structure through hydrophobic interactions and hydrogen bonds, making it effective for controlled-release applications in medications such as aspirin and melatonin [7,8,9,10]. Chitosan is a linear polysaccharide comprising glucosamine and N-acetylglucosamine units, produced by deacetylating chitin. It becomes a cationic polyelectrolyte in acidic environments (pH 6.5) due to the protonation of its amino groups (from -NH_2_ to -NH_3_^+^), forming hydrogels through electrostatic interactions, hydrogen bonds, and hydrophobic interactions, and is used in controlled-release tablets for drugs such as diclofenac sodium and quercetin [11,12,13]. Sodium alginate, derived from algae, forms a rubbery gel in acidic conditions of pH 1–2 via intramolecular hydrogen bonding and expands in higher pH environments due to electrostatic repulsion of deprotonated carboxylate groups (COO^−^). Its ability to interact electrostatically with cationic entities makes it suitable for controlled-release formulations of API like acetylsalicylic acid and mesalazine. Together, these excipients enhance the controlled release and effectiveness of various pharmaceutical products [14,15,16,17,18].

Curcumin, a bioactive compound primarily derived from turmeric (*Curcuma longa* L.) of the ginger family, exhibits a range of significant pharmacological properties. Due to its lipophilic properties, curcumin is predominantly absorbed through the small intestine [19]. This compound demonstrates a diverse spectrum of biological activities, including antibacterial, anti-inflammatory, antioxidant, and wound-healing properties, as well as anticancer effects and potential preventive benefits against Alzheimer’s disease [20,21]. Recent studies have used self-emulsions and liposomes to control the release characteristics and enhance the bioavailability of curcumin [22,23]. However, in past studies, the development of gastro-digestible curcumin tablets reduced the bioavailability. Preventing digestion in the gastric fluid and enhancing the controlled release of curcumin is an important issue [24].

Different excipient formulants have different gelling properties and controlled release behavior, but few studies have evaluated the release characteristics of tablets produced by combined formulants. This study aimed to increase the bioaccessibility of controlled-release CCCTs by investigating and comparing their development with chitosan, sodium alginate, and HPMC using starch as a disintegrant.

## 2. Materials and Methods

### 2.1. Materials

Turmeric extract (95% curcumin content) was purchased from Prakruti Products Pvt. Ltd. (Bengaluru, India). Hypromellose (Metolose^®^, 90SH-4000SR) was purchased from Shin-Etsu Chemical Co., Ltd. (Tokyo, Japan). Chitosan (purity > 99%) was purchased from Charming & Beauty Co. (Taipei, Taiwan). Microcrystalline cellulose and lactose were purchased from Wei Ming Pharmaceutical Co., Ltd. (Taipei, Taiwan). Corn starch (purity > 99%) was purchased from Tai Roun Products Co. (Taipei, Taiwan). Magnesium stearate was purchased from the Showa Chemical Industry Co. (Tokyo, Japan). Other chemical reagents used in this study were all the analytical grades purchased from Sigma-Aldrich Co. (St. Louis, MO, USA).

### 2.2. Preparation of Tablets

#### 2.2.1. Wet Granulation

The method described by Gabbott et al. [25] was used. The turmeric extract (1 g) was extracted in 100 mL of 95% ethanol, poured into an excipient powder, and mixed evenly. A 20-mesh sieve was used to filter the larger particles, and the solution was placed in a hot air drier (Model UE 500; Memmert, Schwabach, Germany) at 55 °C for 24 h.

#### 2.2.2. Tablet Preparation

In the method described by Yuan et al. [1], the lubricant (magnesium stearate) and the particles after wet granulation were evenly mixed. Table 1 lists the tablet formulae that were manufactured using a single-punch tableting machine (Tai-Tzu Machinery Co., New Taipei, Taiwan).

### 2.3. Flowability of Particle Blends

The method described by United States Pharmacopeia (USP) and Mutlu et al. [26] was used in this study. The volume was recorded after 10 and 500 taps (V_10_ and V_500_), and the weight of the particle blends was recorded as W. The bulk density (ρB), tapped density (ρT), Carr’s index, and Hausner ratio of the particle blends were calculated using the following equations:

ρB (g/mL) = W/V_10_ (1)

ρT (g/mL) = W/V_500_ (2)

Carr’s index = (ρT − ρB)/ρT × 100(3)

Hausner ratio = ρT/ρB(4)

### 2.4. Characterization of the Tablets

#### 2.4.1. Weight Variance

The method described in the United States Pharmacopeia (USP) c2091 was followed. Ten tablets were randomly selected using an electronic balance (Model BCE224I-1S; Sartorius AG, Göttingen, Germany) to weigh the total weight and calculate the average weight. Each tablet was weighed separately, and the weight variance of each tablet was calculated.

#### 2.4.2. Friability

The USP c1216 method was used. Ten tablets were randomly selected and measured using a friability tester (Model FT-1, Shin Kwang Machinery Co., New Taipei, Taiwan). Weight changes were measured after 100 drum rotations (25 rpm for 4 min).

#### 2.4.3. Hardness

The USP c1217 method was used. Ten tablets with the same formula were randomly selected and measured using a hardness tester (Model TH-20A; Shin Kwang Machinery Co., New Taipei, Taiwan).

#### 2.4.4. Tensile Strength

Following the method of Berardi et al. [27], the following equation was used to calculate the tensile strength, where H, D, and T represent the hardness (N), diameter (mm), and thickness (mm).

Tensile strength (MPa) = 2H/πDT(5)

#### 2.4.5. Disintegration Time

The USP c701 method was used. A disintegration tester (Model CT-1, New Taipei, Shin Kwang Machinery Co., New Taipei, Taiwan) was used to perform this test. Six tablets containing RO water were randomly selected for the basket-rack assembly. The time taken for complete disintegration of the six tablets was recorded.

#### 2.4.6. Differential Scanning Calorimetry (DSC)

The method described by Chao et al. [28], differential scanning calorimetry (Model DSC 822e, Mettler Toledo Co., Greifensee, Switzerland), was used to measure the thermal properties of the particle blends and tablets. The sample (2.5 ± 0.5 mg) was placed in a 40 μL aluminum crucible. The flow rate of nitrogen gas was 80 mL/min, the heating rate was 10°C/min, and the temperature was increased from 25 °C to 400 °C.

#### 2.4.7. Fourier-Transform Infrared Spectroscopy (FT-IR)

Following the method of Chao et al. [28] we used a Fourier-transform infrared spectrometer (Model Bruker Vertex 70V, Hyperion 3000, 6700 FT-IR ATR, Thermo Fisher Scientific Inc., Waltham, MA, USA) and an MCT IR detector to analyze the sample. The sample and potassium bromide were evenly mixed and then compressed into a tablet for analysis. The resolution was set to 4 cm^−1^ and the sample and background scan times were set to 32 scans. The scan range was 4000–650 cm^−1^.

#### 2.4.8. Release Properties of Curcumin

The USP c711 method was used for this purpose. The release of curcumin from the tablets was determined using a USP dissolution apparatus (Model DT-6; Shin Kwang Machinery Co., New Taipei, Taiwan) with a basket apparatus. The simulated gastrointestinal fluids were prepared as described by Liang et al. [23] and Chao et al. [28]. The digestion of tablets was maintained in simulated gastric fluids (pH 1.2) for 2 h and in simulated intestinal fluids (pH 6.8) for 22 h at 37.0 ± 0.5 °C and 100 rpm. In this study, digestive fluids were sampled regularly, and the same volume of digestive fluids was added to compensate for this.

#### 2.4.9. Concentration of Curcumin

The amount of curcumin released was determined using an HPLC-DAD system. The system comprised a degaser (Model Degasys DG-1310, Uniflows Co., Ltd., Tokyo, Japan), a pump (Model Chromaster 5110, Hitachi, Ltd., Tokyo, Japan), autosampler (Model PN5300, Postnova, Landsberg am Lech, Germany), a Mightysil RP-18 GP column (250 mm × 4.6 mm i.d. × 5 μm) (Kanto Chemical Co., Tokyo, Japan), and a diode array detector (Model L-2450, Hitachi, Ltd., Tokyo, Japan). Following our previous study [23], the mobile phase comprised 2% aqueous acetate solution, acetonitrile, and methanol (45/55/50, *v*/*v*/*v*). The flow rate was set at 1 mL/min, the wavelength detection was set at 428 nm, and the injection volume was 10 µL.

#### 2.4.10. Microstructure

Following the method of Chao et al. [28], the tablets released in the simulated gastrointestinal fluids were removed and dried for 24 h using a freeze dryer (Model BFD 4.5/-50, Firstek, New Taipei, Taiwan). Following the drying process, the tablets were retrieved, and their microstructures were observed using a stereo microscope (Model SMZ 800, Nikon Co., Tokyo, Japan).

### 2.5. Statistical Analysis

All experimental results were analyzed using IBM SPSS Statistics (version 19, IBM Co., New York, NY, USA), and the data were analyzed using Duncan’s multiple range test to determine significant differences. The significance level was set at *p* < 0.05.

## 3. Results and Discussion

### 3.1. Flowability of Particle Blends

The compatibility of the powders is significantly affected by their flowability, which is crucial for determining the appropriateness of a formulation for tablet compression [26]. The lack of adequate flowability can impede the seamless introduction of a powder blend into the mold for tablet compression or give rise to a residual ejection force, causing problems during the tablet demolding process. This can result in tablet breakage or uneven density, ultimately compromising the quality of the final tablets [29]. The flowability results are presented in Table 2. The bulk densities of all formulations varied between 0.34 ± 0.01 and 0.55 ± 0.02 g/cm^3^, whereas the tapped densities ranged from 0.35 ± 0.00 to 0.69 ± 0.02 g/cm^3^. The flow properties of the powders were evaluated by calculating the Carr index and Hausner ratio using the provided densities. The criteria established by Kaleem et al. [30] were employed to assess the flowability of powders.

According to the Carr index classification, Formulae A, B, C, and F were categorized as “excellent (0–10)” which demonstrated superior performance, with Formula A, which consisted of HPMC, demonstrating the highest level of flowability. Formulae D, E, and G were classified as “fair (15–20)” exhibiting commendable flowability. In relation to the Hausner ratio, the classification of the Formulae was as follows: Formulae A, B, C, and F were categorized as “excellent (1.00–1.15)”. Formulae E and G were classified as “fair (1.20–1.25)” and Formula D was designated as “passable (1.25–1.35)”. The findings of this study indicated that formulations containing sodium alginate and corn starch demonstrated inferior flowability, which is consistent with Adebayo et al. [31].

### 3.2. Characterization of the Tablets

**Figure 1:**
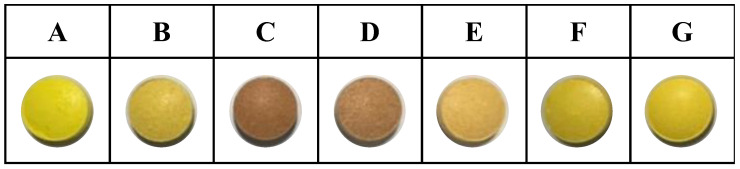
The appearance of combined curcumin controlled-release tablets (**A**–**G**: different tablet formulae).

Figure 1 shows the tablets produced from various formulations of CCCT, and Table 3 summarizes the fundamental characteristics of these tablets. The tablets exhibited a weight variation within the acceptable range of 0.3%, conforming to the United States Pharmacopoeia (USP) standards, which stipulate a maximum weight variation of 2%. The tablets exhibit friability values ranging from 0% to 1.22%, except for Formula E, which experienced tablet breakage during testing. According to the standards set by the USP, the friability of tablet products should not exceed 1%. Based on this criterion, Formula E fails to meet the specified standards. The tablets exhibited hardness values ranging from 5.63 ± 0.25 to 1.98 ± 0.17 kgf. These values conveyed the requirements stipulated by the USP, which mandates a minimum hardness of 4 kgf. Hence, Formulae A, B, and C were in accordance with the established hardness criteria. Formula A exhibited the highest tensile strength, whereas Formula G exhibited the lowest tensile strength. The disintegration duration ranged from 0 to 401 min. Upon contact with the dissolution medium, Formulae C and G disintegrated rapidly. Formula C consisted of chitosan; the structural stability of the corresponding tablets compromised inadequate hydrogen bonding and hydrophobic interactions. Formula G contained corn starch, a substance that swells upon exposure to water. This phenomenon led to the loss of structural integrity and the subsequent disintegration of the tablets. When indicators such as hardness and tensile strength were examined in relation to tablet structural strength, it became apparent that formulations with lower powder flowability tended to display diminished tablet structural integrity [32].

### 3.3. Differential Scanning Calorimetry (DSC)

DSC analysis is a thermal analysis used to characterize the thermal properties of various samples. This technique is used to evaluate the thermal properties of samples and investigate potential interactions between functional components or excipients in tablets after instantaneous compression under high-pressure and high-temperature conditions [33]. A potential compromise in functional component efficacy may arise from the interactions between the raw materials. The presence of new peaks in the DSC thermogram indicates such interactions. In the thermogram, the upward peaks corresponded to exothermic events, whereas the downward peaks indicated endothermic events.

**Figure 2:**
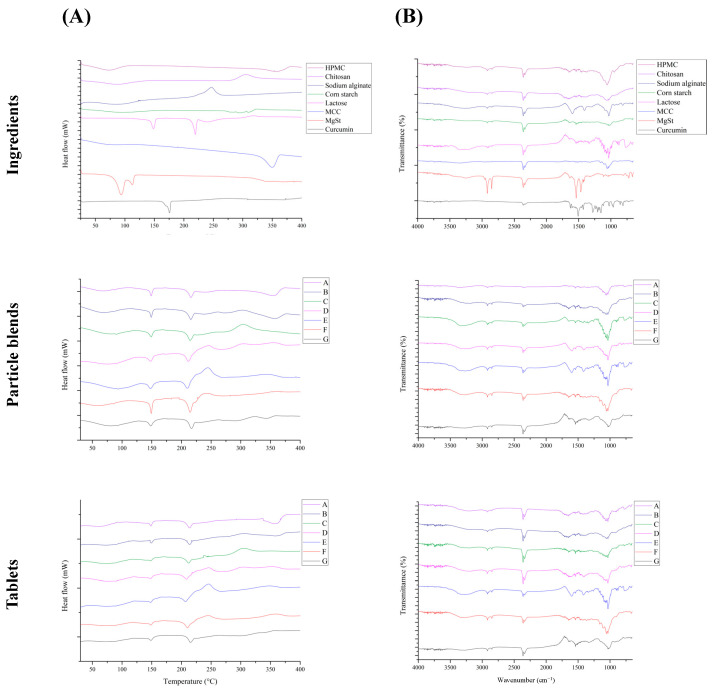
Thermograms and FTIR spectra of ingredients, particle blend, and combined curcumin controlled-release tablets. (**A**) Differential scanning calorimetry; (**B**) Fourier-transform infrared spectroscopy. HPMC: hydroxypropyl methylcellulose, MCC: microcrystalline cellulose, and MgSt: magnesium stearate.

Figure 2 shows thermograms of the ingredients used in this study. HPMC exhibited endothermic peaks at 75 and 366 °C. Thermal analysis of chitosan revealed an endothermic peak at approximately 70 °C, associated with the evaporation of moisture. An additional exothermic peak was observed at approximately 295 °C, which was attributed to the decomposition of the amino groups (-NH_2_) [34]. Thermal analysis of sodium alginate revealed an exothermic peak at approximately 240 °C, which is commonly associated with degradation and the subsequent formation of carbonates [35]. The presence of an endothermic peak at 260 °C in corn starch indicates starch carbonization [36]. Thermal analysis of lactose revealed two distinct endothermic peaks. The first peak, observed at 152.7 °C, was attributed to lactose dehydration. The second peak, occurring at a higher temperature (218.38 °C), signified the melting of lactose and suggested the presence of monohydrate lactose [37]. The thermal decomposition of microcrystalline cellulose is characterized by the presence of an exothermic peak at 350 °C [38]. Thermal analysis of magnesium stearate revealed two distinct endothermic peaks. The first peak observed at 81 °C was attributed to dehydration. The second peak, observed at 120 °C, indicated a phase transition and suggested the presence of dihydrate magnesium stearate [39]. The melting of curcumin crystals produces an endothermic peak at 178 °C in the case of curcumin [40].

Figure 2 shows a comparison of the thermograms of the precompression powder blend and tablets. Upon conducting a comparative analysis between the thermograms of the powder blend before and compression and the thermogram of the powder blend after compression, no additional endothermic or exothermic peaks were observed. This observation suggested that the thermal properties of the excipients and key components did not differ after tablet compression, indicating that no interaction occurred between the excipients and key components within the tablets [37]. These results provide evidence that the excipients used in this study were suitable for CCCTs.

### 3.4. Fourier-Transform Infrared Spectroscopy (FTIR)

Fourier-transform infrared spectroscopy (FTIR) was used to analyze the chemical bonds and functional groups, confirming the interactions that occur during tablet compression [41]. These interactions can lead to additional peaks in the spectrum, indicating the formation of novel chemical bonds. Figure 2 shows the FTIR spectra of the components. The prominent peak observed within the spectral region of 3500–3200 cm^−1^ is indicative of the stretching vibration of the hydroxyl groups (-OH) [42]. The observed peaks within the range of 2830–2695 cm^−1^ can be attributed to the stretching vibrations of the aldehyde functional groups (-CH). The peak observed at 1641 cm^−1^ in the hydroxypropyl methylcellulose (HPMC) spectrum can be attributed to the stretching vibration of the carbonyl groups (C=O) [43]. The spectrum of chitosan exhibits distinct peaks at specific wavenumbers, the peak observed at 1651 cm^−1^ is attributed to the stretching vibrations of the amide (I) groups. Similarly, the peak observed at 1587 cm^−1^ corresponded to the stretching vibration of the amide (II) groups. Finally, the peak at 1380 cm^−1^ can be attributed to the stretching vibration of the methylene groups (-CH_2_) [44]. The presence of a prominent peak at 1595 cm^−1^ in the sodium alginate spectrum suggested the presence of carboxylate groups (-COO^−^) in the alginate compound [45]. The peak observed at 1155 cm^−1^ in the microcrystalline cellulose spectrum indicates the stretching vibration associated with the β-1,4-glycosidic linkage (C-O-C). Similarly, the peak observed at 1064 cm^−1^ corresponds to the stretching vibration of cellulose structures, specifically the C-O or C-C bonds [46]. Within the curcumin spectrum, the presence of a peak at 1626 cm^−1^ signified the existence of a carbon–carbon double bond (C=C). Similarly, the robust peak observed at 1601 cm^−1^ is indicative of a carbon–carbon double bond (C=C) within an aromatic ring. Furthermore, the peak observed at 1508 cm^−1^ correlated with the presence of a carbonyl group (C=O) [47]. The peaks at approximately 2349 cm^−1^ were attributed to the presence of carbon dioxide (O=C=O) within the instrumental background and were independent of the characteristics of the sample under investigation.

### 3.5. Release Properties of Curcumin

**Figure 3:**
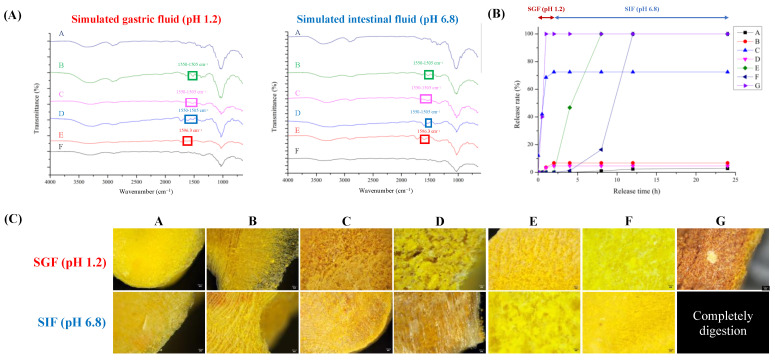
Simulated in vitro simulated gastrointestinal digestion of combined curcumin controlled-release tablets. (**A**) FTIR spectra; (**B**) release rate of curcumin; (**C**) microstructure.

The release profiles of the tablets are shown in Figure 3. Figure 3A,B show the FTIR spectra and release properties of the tablets subjected to simulated gastrointestinal fluids.

Formula A consists of HPMC, a compound that exhibits insensitivity toward changes in the pH level. Moreover, upon hydration, HPMC forms a resilient water–gel structure [11]. The HPMC variant utilized in this investigation possesses an increased viscosity of 4.0 Pa·s, inducing restricted breakdown even after 24 h, consequently yielding diminished curcumin release. The FTIR spectrum showed no discernible changes under either acidic or neutral conditions, proving the stability of the sample over the pH range of 3 to 11.

Formula B combined HPMC and chitosan. Chitosan is particularly soluble in acidic environments, leading to increased release rates within the first 2 h of exposure to simulated gastric fluids and undergoes a transition to a higher pH environment resembling simulated intestinal fluids, resulting in the formation of a gel, which can significantly reduce the release rate (Figure 3B).

Formula C comprises chitosan, which demonstrates accelerated release rates during the initial 2 h conveying its solubility in acidic environments [48]. In the current phase, the amino groups of chitosan undergo protonation in an acidic environment, resulting in the dissolution and disintegration of the compound. When chitosan was exposed to simulated intestinal fluids (SIF) at pH 6.8, it started to gel, decreasing its release rate and limiting disintegration (Figure 3B). The FTIR spectrum exhibited distinct symmetric and asymmetric deformations associated with protonated amino groups of chitosan within the spectral regions 1625–1560 cm^−1^ and 1550–1505 cm^−1^, respectively [49].

Formula D contains chitosan and sodium alginate. The tablets exhibited higher release rates in SIF, which can be attributed to the increased solubility of chitosan. In SIF, the electrostatic interactions between the carboxylate anions (-COO^−^) of sodium alginate and protonated amino groups (-NH_3_^+^) of chitosan resulted in the formation of a polyelectrolyte membrane. The membrane significantly reduces the release rate [50]. The FTIR spectrum demonstrates the absence of distinct peaks associated with protonated amino groups of chitosan within the wavenumber ranges of 1625–1560 cm^−1^ and 1550–1505 cm^−1^, which can be attributed to interactions with carboxylate anions.

Formula E contains sodium alginate. When the pH is lower than the pKa (pH 4), carboxylate anions are transformed into free carboxylate groups, forming alginic acid. Alginic acid does not dissolve under these conditions and forms a stiff, rubbery outer layer. When exposed to SIF (pH 6.8), the tablet forms a swollen, hydrated gel layer on the surface and starts to disintegrate [51]. Thus, the release rate of curcumin increased (Figure 3B). The FTIR spectrum showed no peak at 1590 cm^−1^ under acidic conditions, confirming the synthesis of alginic acid. A peak was observed at approximately 1596.3 cm^−1^ in a pH environment higher than the pKa of sodium alginate. This peak suggests the presence of asymmetric COO^−^ stretching vibrations in sodium alginate [52]. Formula F is a combination of HPMC and sodium alginate. Although HPMC exhibits more robust gel formation, the dissolution of sodium alginate is expected in conditions with higher pH levels, resulting in eventual disintegration and full dissolution of the formulation. When comparing the controlled-release tablets colloidized with HPMC, Formula F exhibited significantly enhanced controlled release compared to Formula A. Formula G consisted of only corn starch and functions as an uncontrolled-release control group. The substance underwent rapid disintegration, resulting in the complete release of curcumin during the first hour [31,36].

### 3.6. Microstructure of Tablet after In Vitro Release Test

The microstructure of the CCCT following the simulated release is shown in Figure 3C. Formulae A, B, and F were incorporated with HPMC and demonstrated the presence of compact network architectures when exposed to simulated gastrointestinal fluids. Formulae containing chitosan and sodium alginate (C and D) form a porous structure in SGF due to the amine groups (-NH_2_) being protonated into -NH_3_^+^, making chitosan positively charged and increasing its solubility and mobility. In SIF, the deprotonation of chitosan and the polyelectrolyte effect of sodium alginate reduces its solubility, forming a stable cross-linked network structure that delays the release of curcumin (Figure 3B). In an acidic environment, sodium alginate forms a strong outer layer (Formula E). Because the carboxyl groups (-COO^−^) were protonated into carboxylic acids (-COOH), their solubility will decrease and promote hydrogel formation. In SIF, because the carboxyl groups remained in a deprotonated state (-COO^−^), a stable hydrogel structure was formed and controlled the release of the curcumin [52]. Formula G consists of starch, which does not form a stable cross-linked structure and is therefore easily disintegrated in both gastrointestinal fluids [53,54]. To sum up, the negatively charged groups of curcumin, such as phenolic hydroxyl or carboxyl groups, are combined with positively charged chitosan through electrostatic interactions in an acidic environment for controlled release. In the CCCT composed of HPMC–sodium alginate (Formula F), the hydroxyl groups of curcumin can form hydrogen bonds with the hydroxyl group, methoxy group, hydroxypropyl group of HPMC, and carboxyl group of sodium alginate. Its hydrogel formation acts as a physical barrier, reducing water penetration and curcumin diffusion. The controlled-release characteristics of CCCT increase the hydrogel structural stability, reduce curcumin diffusibility, and adjust the controlled release of curcumin in simulated gastrointestinal fluids.

## 4. Conclusions

The CCCT with chitosan and HPMC–sodium alginate improved the controlled release characteristics of curcumin during simulated gastric and intestinal digestion, respectively. Compared to starch, the formula with chitosan reduced the release ratio by approximately 27.5% after 2 h in gastric fluid, suggesting its potential as a reference for the development of gastric release tablets. The addition of HPMC strengthened the formation of dense network structures during gastric and intestinal digestion, thereby prolonging the release time from 6 to 12 h. Because of the creation of polyelectrolyte membrane hydrogels and network structures, the formula with HPMC–sodium alginate had a curcumin release rate of less than 20% within 6 h of digestion. This effectively controlled the curcumin released in intestinal fluid. Thus, HPMC–sodium alginate can be an effective strategy for intestinal controlled-release tablet development with the potential to modulate curcumin release. This study provides valuable insights for the future development of healthcare and nutritional curcumin tablets.

## Figures and Tables

**Table 1:** The formulae of combined curcumin controlled-release tablets.

Formulae	Hydroxypropyl Methylcellulose	Chitosan	Sodium Alginate	Corn Starch	Microcrystalline Cellulose	Lactose	Magnesium Stearate
Formulae of Tablets (%)							
A	50	-	-	-	25	24	1
B	25	25	-	-	25	24	1
C	-	50	-	-	25	24	1
D	-	25	25	-	25	24	1
E	-	-	50	-	25	24	1
F	25	-	25	-	25	24	1
G	-	-	-	50	25	24	1

**Table 2:** The flowability of different particle blends.

Formulae	Bulk Density (g/cm^3^)	Tapped Density (g/cm^3^)	Carr Index	Hausner Ratio
A	0.34 ^e^ ± 0.01	0.35 ^e^ ± 0.00	2.92 ^c^ ± 0.92	1.03 ^c^ ± 0.01
B	0.34 ^e^ ± 0.01	0.36 ^e^ ± 0.01	4.02 ^c^ ± 1.22	1.04 ^c^ ± 0.01
C	0.48 ^c^ ± 0.01	0.50 ^c^ ± 0.01	3.56 ^c^ ± 1.17	1.03 ^c^ ± 0.01
D	0.51 ^b^ ± 0.01	0.64 ^b^ ± 0.02	20.73 ^a^ ± 4.52	1.26 ^a^ ± 0.07
E	0.55 ^a^ ± 0.02	0.69 ^a^ ± 0.02	19.62 ^a^ ± 3.06	1.25 ^a^ ± 0.05
F	0.37 ^d^ ± 0.00	0.41 ^d^ ± 0.00	9.91 ^b^ ± 0.98	1.11 ^b^ ± 0.01
G	0.51 ^b^ ± 0.01	0.64 ^b^ ± 0.01	19.64 ^a^ ± 1.34	1.24 ^a^ ± 0.02

Each value is expressed as mean ± standard deviation (n = 10). a–e Means with different letters within the same column differ significantly (*p* < 0.05).

**Table 3:** The properties of combined curcumin controlled-release tablets.

Formulae	Weight Variation (%)	Friability (%)	Hardness (kgf)	Tensile Strength (MPa)	Disintegration Time (min)
A	−0.31 ^d^ ± 0.00	0.11 ^c^ ± 0.01	5.19 ^b^ ± 0.06	0.95 ^a^ ± 0.01	401.25 ^a^ ± 11.00
B	−0.11 ^c^ ± 0.00	0.00 ^d^ ± 0.03	4.02 ^c^ ± 0.08	0.71 ^c^ ± 0.01	155.85 ^b^ ± 5.25
C	0.32 ^a^ ± 0.00	0.00 ^d^ ± 0.00	5.63 ^a^ ± 0.25	0.88 ^b^ ± 0.04	0.00 ^f^ ± 0.00
D	−0.10 ^c^ ± 0.00	0.01 ^d^ ± 0.00	2.30 ^d^ ± 0.36	0.38 ^e^ ± 0.06	41.50 ^e^ ± 2.25
E	0.02 ^b^ ± 0.00	1.22 ^a^ ± 0.23	2.04 ^e^ ± 0.23	0.33 ^f^ ± 0.04	49.53 ^d^ ± 0.60
F	0.01 ^b^ ± 0.00	0.02 ^d^ ± 0.00	3.85 ^c^ ± 0.07	0.69 ^d^ ± 0.01	124.64 ^c^ ± 8.85
G	0.01 ^b^ ± 0.00	0.21 ^b^ ± 0.02	1.98 ^e^ ± 0.17	0.29 ^g^ ± 0.03	0.00 ^f^ ± 0.00

Each value is expressed as mean ± standard deviation (n = 10). a–g Means with different letters within the same column differ significantly (*p* < 0.05).

## Data Availability

The data presented in this study are available on request from the corresponding author.

## References

[1] Yuan K.C., Chiang Y.C., Li P.H., Chiang P.Y. (2024). Physicochemical and release properties of anthocyanin gastric floating tablets colloidized with κ-carrageenan/metal ions. Food Hydrocoll..

[2] Lordan R. (2021). Dietary supplements and nutraceuticals market growth during the coronavirus pandemic–Implications for consumers and regulatory oversight. PharmaNutrition.

[3] Jadhav H.B., Sablani S., Gogate P., Annapure U., Casanova F., Nayik G.A., Alaskar K., Sarwar N., Raina I.A., Ramniwas S. (2023). Factors governing consumers buying behavior concerning nutraceutical product. Food Sci. Nutr..

[4] López-Córdoba A., Matera S., Deladino L., Hoya A., Navarro A., Martino M. (2015). Compressed tablets based on mineral-functionalized starch and co-crystallized sucrose with natural antioxidants. J. Food Eng..

[5] Maderuelo C., Zarzuelo A., Lanao J.M. (2011). Critical factors in the release of drugs from controlled release hydrophilic matrices. J. Control. Release.

[6] Bruneau M., Bennici S., Brendle J., Dutournie P., Limousy L., Pluchon S. (2019). Systems for stimuli-controlled release: Materials and applications. J. Control. Release.

[7] Mašková E., Kubová K., Raimi-Abraham B.T., Vllasaliu D., Vohlídalová E., Turánek J., Mašek J. (2020). Hypromellose–A traditional pharmaceutical excipient with modern applications in oral and oromucosal drug delivery. J. Control. Release.

[8] Lee B.J., Ryu S.G., Cui J.H. (1999). Formulation and release characteristics of hydroxypropyl methylcellulose matrix tablet containing melatonin. Drug Dev. Ind. Pharm..

[9] Heng PW S., Chan L.W., Easterbrook M.G., Li X. (2001). Investigation of the influence of mean HPMC particle size and number of polymer particles on the release of aspirin from swellable hydrophilic matrix tablets. J. Control. Release.

[10] Joshi S.C. (2011). Sol-gel behavior of hydroxypropyl methylcellulose (HPMC) in ionic media including drug release. Materials.

[11] Fu J., Yang F., Guo Z. (2018). The chitosan hydrogels: From structure to function. New J. Chem..

[12] Sabnis S., Rege P., Block L.H. (1997). Use of chitosan in compressed tablets of diclofenac sodium: Inhibition of drug release in an acidic environment. Pharm. Dev. Technol..

[13] Caddeo C., Nácher A., Díez-Sales O., Merino-Sanjuán M., Fadda A.M., Manconi M. (2014). Chitosan–xanthan gum microparticle-based oral tablet for colon-targeted and controlled delivery of quercetin. J. Microencapsul..

[14] Draget K.I., Skjåk-Bræk G., Stokke B.T. (2006). Similarities and differences between alginic acid gels and ionically crosslinked alginate gels. Food Hydrocoll..

[15] Abd El-Ghaffar M., Hashem M., El-Awady M., Rabie A. (2012). pH-sensitive sodium alginate hydrogels for riboflavin controlled release. Carbohydr. Polym..

[16] Draget K.I., Østgaard K., Smidsrød O. (1990). Homogeneous alginate gels: A technical approach. Carbohydr. Polym..

[17] Holte Ø., Onsøyen E., Myrvold R., Karlsen J. (2003). Controlled release of water-soluble drug from directly compressed alginate tablets. Eur. J. Pharm. Sci..

[18] Tuğcu-Demiröz F., Acartürk F., Takka S., Konuş-Boyunağa Ö. (2007). Evaluation of alginate based mesalazine tablets for intestinal drug delivery. Eur. J. Pharm. Biopharm..

[19] Suresh D., Srinivasan K. (2007). Studies on the *in vitro* absorption of spice principles–curcumin, capsaicin and piperine in rat intestines. Food Chem. Toxicol..

[20] Chen M., Du Z.Y., Zheng X., Li D.L., Zhou R.P., Zhang K. (2018). Use of curcumin in diagnosis, prevention, and treatment of Alzheimer’s disease. Neural Regen. Res..

[21] Giordano A., Tommonaro G. (2019). Curcumin and cancer. Nutrients.

[22] Sohn S.I., Priya A., Balasubramaniam B., Muthuramalingam P., Sivasankar C., Selvaraj A., Valliammai A., Jothi R., Pandian S. (2021). Biomedical applications and bioavailability of curcumin—An updated overview. Pharmaceutics.

[23] Liang Y.X., Li P.H., Chiang Y.C., Song H.Y., Lai Y.J., Chiang P.Y. (2023). Assessment of curcumin self-emulsion containing high methoxyl pectin-whey protein complex: Quality stability by thermal, freeze-thaw treatment, and release characteristics. LWT.

[24] Krisanti E.A., Budiatmadjaja M.G., Mulia K. (2021). Formulation and characterization of gastro-retentive floating tablet contained curcuminoids from *Curcuma longa* extracts for treatment of gastric ulcers. AIP Conference Proceedings.

[25] Gabbott I.P., Al Husban F., Reynolds G.K. (2016). The combined effect of wet granulation process parameters and dried granule moisture content on tablet quality attributes. Eur. J. Pharm. Biopharm..

[26] Mutlu C., Koç A., Erbaş M. (2020). Some physical properties and adsorption isotherms of vacuum-dried honey powder with different carrier materials. LWT.

[27] Berardi A., Bauhuber S., Sawafta O., Warnke G. (2021). Alginates as tablet disintegrants: Understanding disintegration mechanisms and defining ranges of applications. Int. J. Pharm..

[28] Chao P.W., Yang K.M., Chiang Y.C., Chiang P.Y. (2022). The formulation and the release of low–methoxyl pectin liquid-core beads containing an emulsion of soybean isoflavones. Food Hydrocoll..

[29] Morin G., Briens L. (2013). The effect of lubricants on powder flowability for pharmaceutical application. AAPS PharmSciTech.

[30] Kaleem M.A., Alam M.Z., Khan M., Jaffery SH I., Rashid B. (2021). An experimental investigation on accuracy of Hausner Ratio and Carr Index of powders in additive manufacturing processes. Met. Powder Rep..

[31] Adebayo S.A., Brown-Myrie E., Itiola O.A. (2008). Comparative disintegrant activities of breadfruit starch and official corn starch. Powder Technol..

[32] Moravkar K.K., Shah D.S., Magar A.G., Bhairav B.A., Korde S.D., Ranch K.M., Chalikwar S.S. (2022). Assessment of pharmaceutical powders flowability and comparative evaluation of lubricants on development of gastro retentive tablets: An application of powder flow tester. J. Drug Deliv. Sci. Technol..

[33] Neto H., Novák C., Matos J. (2009). Thermal analysis and compatibility studies of prednicarbate with excipients used in semi solid pharmaceutical form. J. Therm. Anal. Calorim..

[34] Guinesi L.S., Cavalheiro É.T.G. (2006). The use of DSC curves to determine the acetylation degree of chitin/chitosan samples. Thermochim. Acta.

[35] Pathak T.S., Kim J.S., Lee S.J., Baek D.J., Paeng K.J. (2008). Preparation of alginic acid and metal alginate from algae and their comparative study. J. Polym. Environ..

[36] Liu X., Yu L., Liu H., Chen L., Li L. (2009). Thermal decomposition of corn starch with different amylose/amylopectin ratios in open and sealed systems. Cereal Chem..

[37] Monajjemzadeh F., Hassanzadeh D., Valizadeh H., Siahi-Shadbad M.R., Mojarrad J.S., Robertson T.A., Roberts M.S. (2009). Compatibility studies of acyclovir and lactose in physical mixtures and commercial tablets. Eur. J. Pharm. Biopharm..

[38] Uesu N.Y., Pineda E.A., Hechenleitner A.A. (2000). Microcrystalline cellulose from soybean husk: Effects of solvent treatments on its properties as acetylsalicylic acid carrier. Int. J. Pharm..

[39] Wang T., Potts A.R., Hoag S.W. (2019). Elucidating the variability of magnesium stearate and the correlations with its spectroscopic features. J. Pharm. Sci..

[40] Li J., Shin G.H., Lee I.W., Chen X., Park H.J. (2016). Soluble starch formulated nanocomposite increases water solubility and stability of curcumin. Food Hydrocoll..

[41] Ewing A.V., Biggart G.D., Hale C.R., Clarke G.S., Kazarian S.G. (2015). Comparison of pharmaceutical formulations: ATR-FTIR spectroscopic imaging to study drug-carrier interactions. Int. J. Pharm..

[42] Kasprzyk I., Depciuch J., Grabek-Lejko D., Parlinska-Wojtan M. (2018). FTIR-ATR spectroscopy of pollen and honey as a tool for unifloral honey authentication. The case study of rape honey. Food Control.

[43] Huang H.C., Chen L.C., Lin S.B., Chen H.H. (2011). Nano-biomaterials application: In situ modification of bacterial cellulose structure by adding HPMC during fermentation. Carbohydr. Polym..

[44] Smitha B., Sridhar S., Khan A. (2005). Chitosan–sodium alginate polyion complexes as fuel cell membranes. Eur. Polym. J..

[45] Xiao Q., Gu X., Tan S. (2014). Drying process of sodium alginate films studied by two-dimensional correlation ATR-FTIR spectroscopy. Food Chem..

[46] Hamdan M.A., Ramli N.A., Othman N.A., Amin K.N.M., Adam F. (2021). Characterization and property investigation of microcrystalline cellulose (MCC) and carboxymethyl cellulose (CMC) filler on the carrageenan-based biocomposite film. Mater. Today Proc..

[47] Mohan P.K., Sreelakshmi G., Muraleedharan C., Joseph R. (2012). Water soluble complexes of curcumin with cyclodextrins: Characterization by FT-Raman spectroscopy. Vib. Spectrosc..

[48] Du H., Liu M., Yang X., Zhai G. (2015). The design of pH-sensitive chitosan-based formulations for gastrointestinal delivery. Drug Discov. Today.

[49] Lawrie G., Keen I., Drew B., Chandler-Temple A., Rintoul L., Fredericks P., Grøndahl L. (2007). Interactions between alginate and chitosan biopolymers characterized using FTIR and XPS. Biomacromolecules.

[50] Li L., Wang L., Shao Y., Ni R., Zhang T., Mao S. (2013). Drug release characteristics from chitosan–alginate matrix tablets based on the theory of self-assembled film. Int. J. Pharm..

[51] Hodsdon A.C., Mitchell J.R., Davies M.C., Melia C.D. (1995). Structure and behaviour in hydrophilic matrix controlled release dosage forms: 3. The influence of pH on the controlled-release performance and internal gel structure of sodium alginate matrices. J. Control. Release.

[52] Papageorgiou S.K., Kouvelos E.P., Favvas E.P., Sapalidis A.A., Romanos G.E., Katsaros F.K. (2010). Metal–carboxylate interactions in metal–alginate complexes studied with FTIR spectroscopy. Carbohydr. Res..

[53] Ramírez C., Millon C., Nunez H., Pinto M., Valencia P., Acevedo C., Simpson R. (2015). Study of effect of sodium alginate on potato starch digestibility during in vitro digestion. Food Hydrocoll..

[54] Baysal G., Olcay H.S., Günneç Ç (2023). Encapsulation and antibacterial studies of goji berry and garlic extract in the biodegradable chitosan. J. Bioact. Compat. Polym..

